# Tissue microarrays characterise the clinical significance of a VEGF-A protein expression signature in gastrointestinal stromal tumours

**DOI:** 10.1038/sj.bjc.6603551

**Published:** 2007-02-13

**Authors:** M Salto-Tellez, M E Nga, H C Han, A S-C Wong, C K Lee, D Anuar, S S Ng, M Ho, A Wee, Y H Chan, R Soong

**Affiliations:** 1 Department of Pathology, Yong Loo Lin Faculty of Medicine, National University of Singapore, 5 Lower Kent Ridge Road, Singapore 119074; 2 Oncology Research Institute, National University of Singapore, Level 5 CRC Building, MD11, 10 Medical Drive, Singapore 117597; 3 Department of Hematology Oncology, National University Hospital, 5 Lower Kent Ridge Road, Singapore 119074; 4 Biostatistics Unit, Yong Loo Lin Faculty of Medicine, National University of Singapore, CRC Building, MD11, 10 Medical Drive, Singapore 117597

**Keywords:** gastrointestinal stromal tumours, VEGF, tissue microarrays

## Abstract

A tissue microarray analysis of 22 proteins in gastrointestinal stromal tumours (GIST), followed by an unsupervised, hierarchical monothetic cluster statistical analysis of the results, allowed us to detect a *vascular endothelial growth factor (VEGF) protein overexpression signature* discriminator of prognosis in GIST, and discover novel *VEGF-A* DNA variants that may have functional significance.

The clinical behaviour of gastrointestinal stromal tumours (GIST) is notoriously difficult to predict. The prognostic and therapeutic significance of KIT mutations is somewhat contradictory (Ernst *et al*, 1998; Lasota *et al*, 1999; Moskaluk *et al*, 1999; Taniguchi *et al*, 1999; Lasota *et al*, 2000; Hirota *et al*, 2001; Wardelmann *et al*, 2002; Koay *et al*, 2005). Therefore, it appears that new molecular indicators of prognostication are needed. Tissue microarrays (TMA) is a high-throughput method for the analysis of large numbers of formalin-fixed, paraffin-embedded (FFPE) materials with minimum cost and effort (Kononen *et al*, 1998). Here, we applied the TMA technology to analyse protein expression in GIST. The results were analysed with an unsupervised, hierarchical monothetic cluster statistical method. Those biomarkers with strong clinical significance were tested for mutation status by both PCR-denaturing high performance liquid chromatography (DHPLC) and direct sequencing. By doing so, we identified a *VEGF-A protein overexpression signature* as a statistically significant predictor of malignancy, discovered *VEGF-A* ligand DNA variants in GIST, and provided other possible targets in future design of anti-VEGF-directed therapy against GIST.

## MATERIALS AND METHODS

We used 50 archival paraffin blocks (Department of Pathology, National University Hospital, Singapore), including 15 cases of GIST with a benign outcome, 17 with a malignant outcome (13 primary neoplasms and four metastases), 10 with no available clinical follow-up, and eight gastrointestinal mesenchymal neoplasms other than GIST, such as leiomyoma (*n*=5), leiomyosarcoma, neurofibroma and schwannoma (one of each). The mean clinical follow-up was of 39 months. The overall clinico-pathological characteristics are summarised in Table 3. No chemo or radiotherapy was given to these patients. All the gross and histopathological parameters classically associated with malignant potential were analysed. The findings were similar to those reported in other series (data not shown) and, in themselves, are considered insufficient for single-case prognostication in the clinical setting.

After case review for diagnostic confirmation, the TMA was constructed as reported elsewhere (Zhang *et al*, 2003a; Salto-Tellez *et al*, 2004). The 22 antibodies used are 34 BE12, AE 1/3, Bcl-2, CAM 5.2, CD10, CD117, CD34, c-erbB2, CK7, CK20, Desmin, Flk-1, Flt-1, Hep Par1, Ki-67, MNF 116, p53, PCNA, S100, SMA, VEGF-A and Vimentin. Table 4 indicates the antibodies and their technical specifications. In general, these antibodies can be divided into several groups: diagnostic markers, antibodies expressed in a specific differentiation pathway relevant to GIST, proliferative or apoptosis-related markers, angiogenic proteins, and others that may have been associated before with prognostic significance in GIST. The interpretation of the IHC staining results for TMA was confirmed by three independent observers (NME, LCK and MST). Results were interpreted based on previous published experience for each individual antibody.

The concordance between TMA and full sections, tested for five antibodies (Table 5) ranged from 92–100% in five of six antibodies, excluding S100 (71%), in concordance with previous published results (Zhang *et al*, 2003a).

The 28 FFPE cases with available clinical follow-up were the subject of genomic DNA extraction (GENTRA DNA Purification Kit – Gentra, Minneapolis, MN, USA), according to the manufacturer's instruction. Mutation analysis was performed by PCR-DHPLC analysis. Briefly, DNA was amplified in 25 *μ*l reactions containing 2 *μ*l DNA template, 1 *μ*l of each forward and reverse primers (10 *μ*M each), 0.5 *μ*l of 10 mM dNTP, 0.2 *μ*l FastStart Taq (Roche, Mannheim, Germany), and 1 × PCR reaction buffer with MgCl_2_. Primer sequences and cycling conditions are indicated in Tables 6 and 7. The PCR product (8 *μ*l) was denatured at 95°C for 5 min followed by gradual re-annealing to room temperature for over a period of 1 h. DHPLC was performed using a fully automated WAVE 3500HT system (Transgenomic, Omaha, NE, USA). The cooled samples were automatically injected into a DNASep cartridge (Transgenomic) and eluted at a flow rate of 0.9 ml min^−1^ through a linear gradient of acetonitrile containing 0.1 M triethylammonium acetate (TEAA). Buffer A (0.1 M TEAA solution) and buffer B (0.1 M TEAA with 25% acetonitrile solution) concentrations and oven temperatures for optimal heteroduplex separation under partially DNA denaturation was determined using the WAVE Navigator software followed by empirical adjustment. Amplicons from the HeLa cell line were included in each run as a wild-type reference.

Samples showing a dHPLC aberrant elution profile were re-amplified and sequenced in both directions. Direct sequencing was performed on the ABI PRISM Model 3100 DNA sequencer (Applied Biosystems, Foster City, CA, USA), using the same primers as were used for amplification. Sequencing reactions were conducted with the ABI PRISM BigDye Terminator v3.1 Cycle Sequencing Kit (Applied Biosystems) according to the manufacturer's instructions.

The monothetic cluster analysis was carried out as reported elsewhere (Zhang *et al*, 2003b) Significance tests included the student's unpaired *t*-test (2-tailed) for numerical variables and the Fisher's exact probability test for categorical variables. Significance value for *P* was taken to be *P*<0.05.

## RESULTS

### VEGF protein expression signature and its prognostic significance in GIST

**Figure 1:**
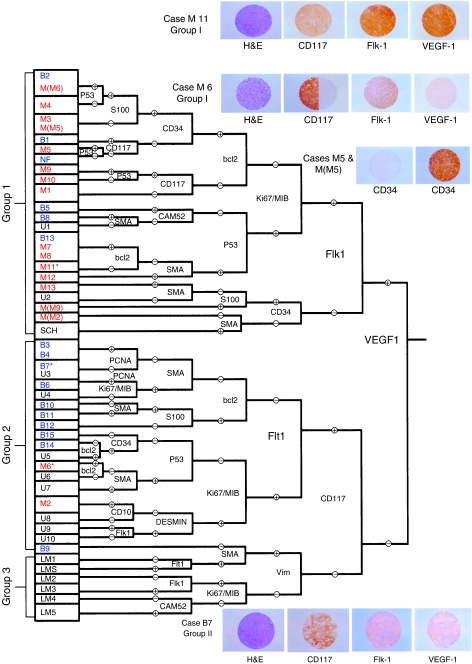
In red are the study cases with malignant behaviour, in blue are those cases with benign behaviour; cases without available follow-up and non-GISTs are in black. The TMA immunohistochemistry results are included. The asterisk indicates cases reflected in the photomicrographs. Other abbreviations are similar to those described in Table 1. VEGF1 is equivalent to VEGF-A in this figure.

Table 1 shows the whole protein expression results. Figure 1 shows the cluster diagram obtained upon monothetic hierarchical cluster analysis, including IHC of representative cases. From the cluster analysis, two main groups emerged, based on reactivity for the VEGF-A ligand antibody. Group 1 includes all the VEGF-A ligand expressing cases; out of the 20 GISTs with known clinical outcome, 15 were malignant (75%). In group 2 (VEGF-A negative), only 2/11 of the cases (18%) had a malignant outcome. The difference was statistically significant (*P=*0.003). Within group 2, the two malignant cases are further subclassified into a cluster arm, which is positive for flt-1, a receptor for VEGF. Hence, all 17/17 malignant cases were positive for either VEGF-A ligand or the VEGF-A receptor, flt-1, as compared to 8/15 of the benign cases (*P=*0.002). In all, 13/17 malignant cases were positive for *both* these markers as compared to 4/15 benign cases (*P=*0.006). Indeed, concomitant expression of VEGF ligand and VEGF receptor represents a *VEGF-A protein expression signature* in GIST with obvious clinical significance. Lastly, proliferation and oncogenic-related markers PCNA, Ki-67/MIB, bcl-2 and p53 showed no statistically significant preference in reactivity for malignant GISTs (*P>0.05*). The fact that all the smooth muscle lesions included in the analysis are clustering in a separate group (group 3) is a measure of the robustness of this analytical approach.

### New VEGF-A variants are discovered as a result of mutation analysis

**Figure 2:**
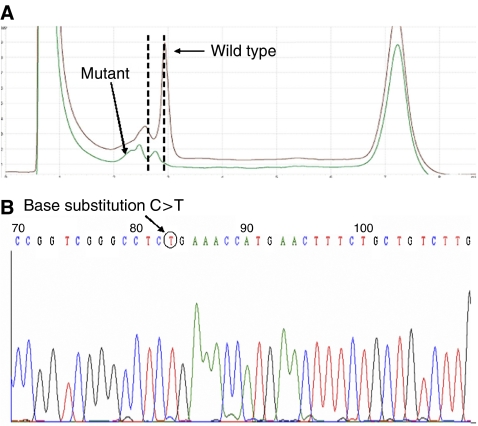
(**A**) DHPLC analysis of *VEGF-A* ligand exon 1: the mutant has an additional peak (indicated by the arrow) and shows a shift in elution time (indicated by the vertical hashed lines). (**B**) Sequencing chromatogram of *VEGF-A* ligand exon 1: direct sequencing indicated that the mutation in the sample is an IVS1 −7C>T variant.

**Figure 3:**
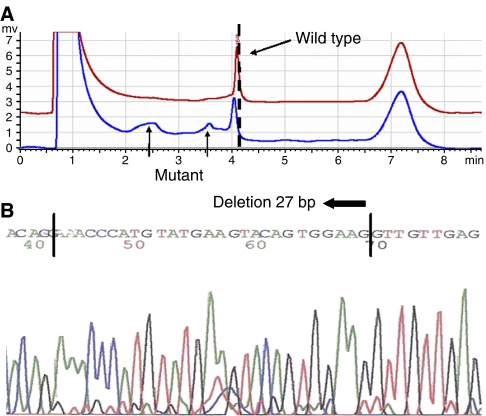
(**A**) DHPLC analysis of *KIT* exon 11: the mutant has two additional peaks (indicated by the arrows) and shows a mild shift in elution time (indicated by the vertical hashed lines). (**B**) Sequencing chromatogram of *KIT* exon11: direct sequencing indicated that the mutation in the sample is a 27 bp deletion.

Those GIST samples with known clinical follow-up underwent genomic analysis. In view of the evidence of *KIT* mutations in GIST and their possible prognostic value (as well as their relation to imatinib therapeutic response) (Lasota *et al*, 1999; Heinrich *et al*, 2003), exons 9, 11, 13, 17 of *KIT* (which are those related to prognosis in the literature) were analysed in the same methodological manner. The results are summarised in Table 2. Variants identified included non-coding IVS1–7:C → T changes in five (18%) samples (Figure 2), IVS4–28:C → T changes in seven (25%) samples and coding codon 48A:G → T (Q → H) and codon 91A:G → A (G → D) changes in one sample each (Table 2). A total of 12 (43%) cases had variants in *KIT*, all in exon 11 (Figure 3). *VEGF* IVS4–28:C → T variants were more frequent in samples with low (5/7, 71%) than high (2/7, 29%) VEGF-A expression. The *VEGF* codon 48 and 91 mutants were present in samples with high VEGF-A expression, *KIT* mutations and of a malignant phenotype. *KIT* mutations were more frequent in samples with high (10/22, 46%) than low (2/6, 21%) KIT expression. Nevertheless, none of the associations between sequence variants and the expression of their respective proteins, or with the presence of each other, were significant, presumably due to the limited number of samples in this series. The only parameter significantly associated with malignancy in these selected 28 cases was, as expected, VEGF-A protein expression (*P=*0.020). Of interest, there was no association with exon 11 *KIT* mutations and survival in our series.

## DISCUSSION

The uncertain prognosis of GIST, both before (Nilsson *et al*, 2005) and after (Kosmadakis *et al*, 2005) imatinib treatment, indicate the need for the search of other molecular prognostication biomarkers.

GIST are highly vascularised neoplasms and VEGF-A is a major antiangiogenic therapeutic target (Ferrara and Kerbel, 2005). Recently, anti-VEGF-A therapy has been successful in the treatment of GIST (Marx 2005), with drugs such as Sutent and Sorafenib. Our results indicate that a combined VEGF-A ligand-receptor protein expression signature is a determinant of clinical behaviour in GIST. This is obvious in our study because (a) there is a relation between protein overexpression of the VEGF-A ligand and Flt-1 proteins and benign/malignant behaviour; (b) novel variants in the *VEGF-A ligand* gene are characterised, some of which appear related to a malignant behaviour (such as *VEGF-A* exon 3); and (c) in general, these VEGF-ligand variants localise to areas of the VEGF protein with functional significance. The role of flt-1 in this context is unclear; it could be related to the induction of metalloproteinases (Hiratsuka *et al*, 2002), or to chemotactic signals (Wey *et al*, 2005).

The role of the detected VEGF-A ligand variants in protein overexpression and GIST tumorigenesis can only be a matter of speculation, based on the scant information available. The IVS4–28:C → T variant is also identified in phenotypically normal gastrointestinal tissue, thus may not be relevant. The two other variants, however, may have functional implications. The IVS1–7:C → T variant lies within a GC box that binds the transcriptional repressor protein methyl CpG binding protein-2 (Lapchak *et al*, 2004), and was found to be associated with higher levels of VEGF mRNA in colorectal cancer (Yamamori *et al*, 2004), increasing the risk of liver metastasis and worsening its prognosis. In addition, two missense mutations (unreported to date) were discovered in exon 3, coding codon 48A:G → T (Q → H) and codon 91A:G → A (G → D), both in malignant GIST and both showing VEGF-A ligand protein overexpression. In any case, the evidence points to the novel hypothesis that *VEGF-A* ligand mutations may play a role if the biology and prognosis of GIST.

There has been a previous suggestion that VEGF-A ligand protein expression may be related to prognosis (Takahashi *et al*, 2003). However, the strength of our unsupervised hierarchical cluster analysis, comparing the expression of an antibody in the context of another 21 biomarkers, delineates ‘biological groups’ and establishes more complete ‘prognostic signatures’, which in our study, shown the importance of including protein expression of both VEGF-A ligand and flt-1 receptor in the characterisation of malignant behaviour.

## Figures and Tables

**Table 1:**
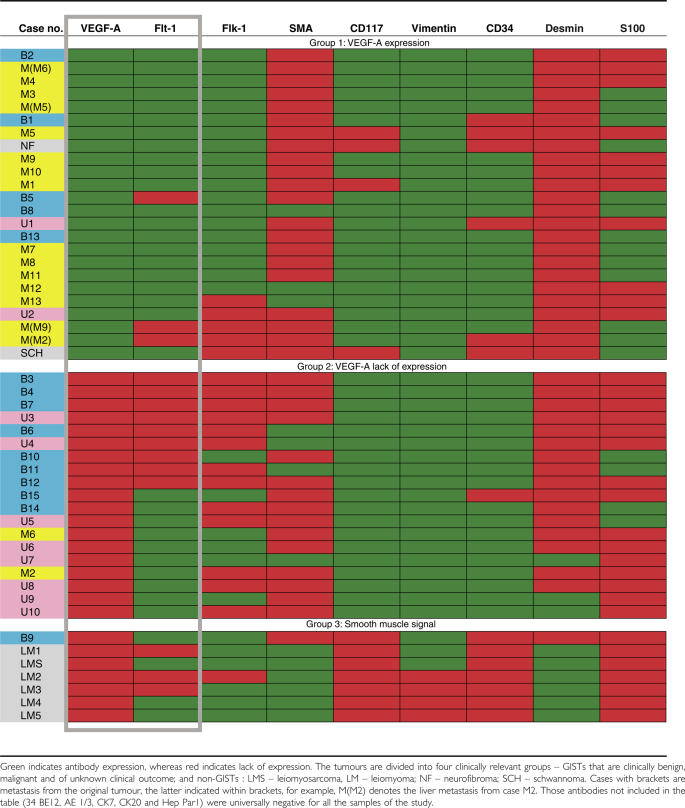
Indication of the immunohistochemistry results based on the groups from the hierarchical cluster analysis (see Figure 1), and highlighting the VEGF protein expression signature

**Table 2:** Protein expression and sequence status of VEGF and KIT in malignant and benign GIST samples

	**VEGF**	**KIT**	**VEGF**	**VEGF**	**VEGF**	**KIT**
**Case**	**IHC**	**IHC**	**Exon 1**	**Exon 3**	**Exon 4**	**Exon 11**
benign						
1	+	+				
2	+	+				550A:deletion 27bp
3	+	+	IVS1–7:C>T			
4	+	+				
5	−	+			IVS4–28:C>T	559C:deletion 6bp
6	−	+			IVS4–28:C>T	572A: insertion 5bp
7	−	+			IVS4–28:C>T	
8	−	+				558A:deletion 9bp
9	−	+				
10	−	+				
11	−	+				
12	−	−	IVS1–7:C>T			
13	−	−			IVS4–28:C>T	
						
malignant						
1	+	+	IVS1–7:C>T			
2	+	+		91A:G>A(G>D)		557A:deletion 6bp
3	+	+			IVS4–28:C>T	550A:deletion 27bp
4	+	+				550A:deletion 27bp
5	+	+				557T:deletion 6bp
6	+	+				558G:deletion 3bp
7	+	+				
8	+	+				
9	+	+				
10	+	+				
11	+	−				
12	+	−	IVS1–7:C>T	48A:G>T(Q>H)	IVS4–28:C>T	550A:deletion 27bp
13	−	+			IVS4–28:C>T	550A:deletion 27bp
14	−	−	IVS1–7:C>T			551C:deletion 12bp
15	−	−				

+=expression, −=no expression.

Sequence variants are denoted as ‘codon followed by nucleotide position (A=1st, B=2nd, C=3rd): nucleotide change (protein change)’. Non-coding variants are denoted as ‘IVS, exon, nucleotides from exon start: nucleotide change’.

**Table 3:** Characteristics of benign (B) and malignant (M) GISTs

**No**	**Age**	**Site**	**Size (mm)**	**Cell type**	**Mitoses (/50 HPF)**	**SMA % +ve**	**CD34 % +ve**	**CD117 % +ve**	**Status (months)**	**Metastases/recurrence**
B1	45	Duod	20	s	2	0	0	100	aned (76)	Nil
B2	39	Gastric	70	m	1.5	0	100	80	aned (124)	Nil
B3	45	Gastric	10	s	0	0	100	95	aned (24)	Nil
B4	46	Gastric	27	s	1	0	100	100	aned (20)	Nil
B5	53	Gastric	29	m	0	0	80	85	aned (24)	Nil
B6	69	Gastric	35	s	1	10	100	40	aned (87)	Nil
B7	71	Gastric	90	s	1	0	100	100	aned (3)	Nil
B8	77	Gastric	45	s	1	0	100	80	aned (68)	Nil
B9	42	Gastric	50	s	3.5	0	0	0	aned (13)	Nil
B10	50	Gastric	100	s	1	0	90	30	aned (10)	Nil
B11	62	Gastric	6	s	1	15	100	100	aned (1)	Nil
B12	87	Gastric	25	s	0	0	100	100	aned (6)	Nil
B13	87	Gastric	7	s	3	0	100	100	aned (12)	Nil
B14	47	Pelvic	60	s	4	0	100	100	aned (83)	Nil
B15	49	Jejunal	45	s	2	0	0	100	aned (60)	Nil
M1	67	Colon	90	s	15	0	100	0	dod (21)	LR
M2	37	Duodenal	60	m	4.5	0	70	50	awd (89)	Liver
M3	36	Gastric	180	s	62.5	0	100	100	dod (17)	Liver
M4	52	Gastric	190	s	7.5	0	100	100	dod (36)	No data
M5	59	Gastric	70	e	10	0	0	0	dod (72)	Liver, bones, abdominal nodes
M6	71	Gastric	170	e	24	0	100	100	awd (103).	Omentum, LR
M7	41	Gastric	100	e	26	0	100	75	dod (43)	Retroperitoneum
M8	48	Gastric	35	s	24.5	0	100	70	dod (27)	Peritoneum
M9	48	Gastric	150	s	31	0	100	100	dod (22)	Liver, spleen
M10	68	Gastric	110	s	113.5	0	100	85	dod (7)	Liver, LR
M11	73	Gastric	60	s	66.5	0	100	100	dod (8)	No data
M12	65	Jejuno-ileal	90	s	52	45	100	100	awd (15)	Peritoneum
M13	33	Rectal	60	s	0.5	2.5	100	70	duc	Liver, bone, para-aortic nodes, lungs

B=Benign cases; M=Malignant cases; s=spindle cell type; e epithelioid cell type; m=mixed epithelioid and spindle cell type; aned=alive with no evidence of disease; awd=alive with disease; dod=died of disease; duc=died of unrelated causes; LR=local recurrence.

**Table 4:** Antibodies used

**Antibody**	**Type**	**Source**	**Dilution**
34 BE12	Monoclonal	Dako, Glostrup, Denmark	1:500
AE 1/3	Monoclonal	Dako, Glostrup, Denmark	1:1000
Bcl-2	Monoclonal	Dako, Glostrup, Denmark	1:200
CAM 5.2	Monoclonal	Becton-Dickinson, San Jose, CA, USA	1:20
CD10	Monoclonal	Novocastra, Newcastle, UK	1:200
CD117	Polyclonal	Dako, Denmark	1:1000
CD34	Monoclonal	Dako, Glostrup, Denmark	1:1000
c-erbB2	Monoclonal	Signet Laboratories Inc., Dedham, MA, USA	1:200
CK7	Monoclonal	Dako, Glostrup, Denmark	1:2000
CK20	Monoclonal	Neomarker, Fremont, CA, USA	1:200
Desmin	Monoclonal	Neomarker, Fremont, CA, USA	1:500
Flk-1	Monoclonal	Santa Cruz Biotechnology, Santa Cruz, CA, USA	1:500
Flt-1	Monoclonal	Santa Cruz Biotechnology, Santa Cruz, CA, USA	1:1000
Hep Par1	Monoclonal	Dako, Glostrup, Denmark	1:500
Ki-67	Monoclonal	Dako, Glostrup, Denmark	1:100
MNF 116	Monoclonal	Dako, Glostrup, Denmark	1:500
p53	Monoclonal	Dako, Glostrup, Denmark	1:500
PCNA	Monoclonal	Dako, Glostrup, Denmark	1:1000
S100	Polyclonal	Dako, Glostrup, Denmark	1:10 000
SMA	Monoclonal	Dako, Glostrup, Denmark	1:1000
VEGF-A	Monoclonal	Santa Cruz Biotechnology, Santa Cruz, CA, USA	1:500
Vimentin	Monoclonal	Dako, Glostrup, Denmark	1:1000

**Table 5:** Comparison of results of TMA *vs* full section analysis

		**SMA**	**Vim**	**CAM5.2**	**CD117**	**CD34**	**S100**
Full sections	+	14	47	3	39	37	17
	−	37	4	48	12	14	34
TMA	+	14	47	1	35	34	4
	−	37	4	50	16	17	47
Disagree	4	0	2	4	3	15	
Concordance %	92	100	96	92	94	71	

**Table 6:** *KIT* PCR conditions

**Exon**	**Forward primer**	**Reverse primer**	**Size (bp)**	**Tm (°C)**	**DHPLC temperature (°C)**	**DHPLC gradient**
9	5′ATGCTCTGCTTCTGTACTGCC3′	′CAGAGCCTAAACATCCCCTTA3′	185	60	57	47.5–61.5%B in 4.5 min
11	5′CCAGAGTGCTCTAATGACTG3′	5′ACCCAAAAAGGTGACATGGA3′	184	60	56	47.5–61.5%B in 4.5 min
13	5′CATCAGTTTGCCAGTTGTGC3′	5′ACACGGCTTTACCTCCAATG3′	142	60	59	44.2–58.2%B in 4.5 min
17	5′TGTATTCACAGAGACTTGGC3′	5′GGATTTACATTATGAAAGTCACAGG3′	172	55	56	46.7–60.7%B in 4.5 min

**Table 7:** *VEGF-A* PCR conditions

**Exon**	**Forward primer**	**Reverse primer**	**Size (bp)**	**Temperature (°C)**	**Oven temperature (°C)**	**Buffer concentration (%B)**
1	GGGGAGGAAGAGTAGCTCG	GCACCTAAGACGACAGAGGG	324	60	66.8	55.4
2	CTGTTGGTGGGAGGGAAGTG	AAGGAATTAGGCCATCCACC	224	65	63.0	47
3	GCTAGCCATCTTTTGTGTCG	TGTTCCCAAAGTGTTACCCC	314	65	61.8	55.1
4	GGTTGTCCCATCTGGGTATG	TAACCCTGGCACAGATCAGG	210	65	60.9	46.3
5	TCACCATCTTAACCCTTCCC	ACAGAGGTAGCCAAGAGCCC	161	65	60.7	39
6	CCTGCCCACCTTACCACTTC	GAGGCTCCAGGGCATTAGAC	188	65	60.8	41
7	CAGCTGCGGACATGTTAGG	TCGCTCGCTCACTCTCTTTC	313	65	59.8	55.1

## References

[bib1] Ernst SI, Hubbs AE, Przygodzki RM, Emory TS, Sobin LH, O'Leary TJ (1998) KIT mutation portends poor prognosis in gastrointestinal stromal/smooth muscle tumors. Lab Invest 78: 1633–1636 9881963

[bib2] Ferrara N, Kerbel RS (2005) Angiogenesis as a therapeutic target. Nature 438: 967–974 16355214 10.1038/nature04483

[bib3] Heinrich MC, Corless CL, Demetri GD, Blanke CD, von Mehren M, Joensuu H, McGreevey LS, Chen CJ, Van den Abbeele AD, Druker BJ, Kiese B, Eisenberg B, Roberts PJ, Singer S, Fletcher CD, Silberman S, Dimitrijevic S, Fletcher JA (2003) Kinase mutations and imatinib response in patients with metastatic gastrointestinal stromal tumor. J Clin Oncol 21: 4342–4349 14645423 10.1200/JCO.2003.04.190

[bib4] Hiratsuka S, Nakamura K, Iwai S, Murakami M, Itoh T, Kijima H, Shipley JM, Senior RM, Shibuya M (2002) MMP9 induction by vascular endothelial growth factor receptor-1 is involved in lung-specific metastasis. Cancer Cell 2: 289–300 12398893 10.1016/s1535-6108(02)00153-8

[bib5] Hirota S, Nishida T, Isozaki K, Taniguchi M, Nakamura J, Okazaki T, Kitamura Y (2001) Gain-of-function mutation at the extracellular domain of KIT in gastrointestinal stromal tumours. J Pathol 193: 505–510 11276010 10.1002/1096-9896(2000)9999:9999<::AID-PATH818>3.0.CO;2-E

[bib6] Koay MH, Goh YW, Iacopetta B, Grieu F, Segal A, Sterrett GF, Platten M, Spagnolo DV (2005) Gastrointestinal stromal tumours (GISTs): a clinicopathological and molecular study of 66 cases. Pathology 37: 22–31 15875730 10.1080/00313020400023628

[bib7] Kononen J, Bubendorf L, Kallioniemi A, Barlund M, Schraml P, Leighton S, Torhorst J, Mihatsch MJ, Sauter G, Kallioniemi OP (1998) Tissue microarrays for high-throughput molecular profiling of tumor specimens. Nat Med 4: 844–847 9662379 10.1038/nm0798-844

[bib8] Kosmadakis N, Visvardis EE, Kartsaklis P, Tsimara M, Chatziantoniou A, Panopoulos I, Erato P, Capsambelis P (2005) The role of surgery in the management of gastrointestinal stromal tumors (GISTs) in the era of imatinib mesylate effectiveness. Surg Oncol 2: 75–84 10.1016/j.suronc.2005.05.002 15993051

[bib9] Lapchak PH, Melter M, Pal S, Flaxenburg JA, Geehan C, Frank MH, Mukhopadhyay D, Briscoe DM (2004) CD40-induced transcriptional activation of vascular endothelial growth factor involves a 68-bp region of the promoter containing a CpG island. Am J Physiol Renal Physiol 287: F512–F520 15140761 10.1152/ajprenal.00070.2004

[bib10] Lasota J, Jasinski M, Sarlomo-Rikala M, Miettinen M (1999) Mutations in exon 11 of c-Kit occur preferentially in malignant versus benign gastrointestinal stromal tumors and do not occur in leiomyomas or leiomyosarcomas. Am J Pathol 154: 53–60 9916918 10.1016/S0002-9440(10)65250-9 PMC1853448

[bib11] Lasota J, Wozniak A, Sarlomo-Rikala M, Rys J, Kordek R, Nassar A, Sobin LH, Miettinen M (2000) Mutations in exons 9 and 13 of KIT gene are rare events in gastrointestinal stromal tumors. A study of 200 cases. Am J Pathol 157: 1091–1095 11021812 10.1016/S0002-9440(10)64623-8 PMC1850182

[bib12] Marx J (2005) Medicine. Cancer-suppressing enzyme adds a link to type 2 diabetes. Science 310: 1259 16311305 10.1126/science.310.5752.1259a

[bib13] Moskaluk CA, Tian Q, Marshall CR, Rumpel CA, Franquemont DW, Frierson Jr HF (1999) Mutations of c-kit JM domain are found in a minority of human gastrointestinal stromal tumors. Oncogene 18: 1897–1902 10086344 10.1038/sj.onc.1202496

[bib14] Nilsson B, Bumming P, Meis-Kindblom JM, Oden A, Dortok A, Gustavsson B, Sablinska K, Kindblom LG (2005) Gastrointestinal stromal tumors: the incidence, prevalence, clinical course, and prognostication in the preimatinib mesylate era – a population-based study in western Sweden. Cancer 103: 821–829 15648083 10.1002/cncr.20862

[bib15] Salto-Tellez M, Lee SC, Chiu LL, Lee CK, Yong MC, Koay ES (2004) Microsatellite Instability in colorectal cancer-considerations for molecular diagnosis and high-throughput screening of archival tissues. Clin Chem 50: 1082–1086 15161730 10.1373/clinchem.2003.030700

[bib16] Takahashi R, Tanaka S, Kitadai Y, Sumii M, Yoshihara M, Haruma K, Chayama K (2003) Expression of vascular endothelial growth factor and angiogenesis in gastrointestinal stromal tumor of the stomach. Oncology 64: 266–274 12697968 10.1159/000069316

[bib17] Taniguchi M, Nishida T, Hirota S, Isozaki K, Ito T, Nomura T, Matsuda H, Kitamura Y (1999) Effect of c-kit mutation on prognosis of gastrointestinal stromal tumors. Cancer Res 59: 4297–4300 10485475

[bib18] Wardelmann E, Neidt I, Bierhoff E, Speidel N, Manegold C, Fischer HP, Pfeifer U, Pietsch T (2002) c-kit mutations in gastrointestinal stromal tumors occur preferentially in the spindle rather than in the epithelioid cell variant. Mod Pathol 15: 125–136 11850541 10.1038/modpathol.3880504

[bib19] Wey JS, Fan F, Gray MJ, Bauer TW, McCarty MF, Somcio R, Liu W, Evans DB, Wu Y, Hicklin DJ, Ellis LM (2005) Vascular endothelial growth factor receptor-1 promotes migration and invasion in pancreatic carcinoma cell lines. Cancer 104: 427–438 15952180 10.1002/cncr.21145

[bib20] Yamamori M, Sakaeda T, Nakamura T, Okamura N, Tamura T, Aoyama N, Kamigaki T, Ohno M, Shirakawa T, Gotoh A, Kuroda Y, Matsuo M, Kasuga M, Okumura K (2004) Association of VEGF genotype with mRNA level in colorectal adenocarcinomas. Biochem Biophys Res Commun 325: 144–150 15522212 10.1016/j.bbrc.2004.10.005

[bib21] Zhang D, Salto-Tellez M, Putti TC, Do E, Koay ES (2003a) Reliability of tissue microarrays in detecting protein expression and gene amplification in breast cancer. Mod Pathol 16: 79–85 12527717 10.1097/01.MP.0000047307.96344.93

[bib22] Zhang DH, Salto-Tellez M, Chiu LL, Shen L, Koay ES (2003b) Tissue microarray study for classification of breast tumors. Life Sciences 73: 3189–3199 14561524 10.1016/j.lfs.2003.05.006

